# Association between fluid management and dilutional coagulopathy in severe postpartum haemorrhage: a nationwide retrospective cohort study

**DOI:** 10.1186/s12884-018-2021-9

**Published:** 2018-10-11

**Authors:** Ada Gillissen, Thomas van den Akker, Camila Caram-Deelder, Dacia D C A Henriquez, Kitty W M Bloemenkamp, Jos J M van Roosmalen, Jeroen Eikenboom, Johanna G van der Bom, on behalf of the TeMpOH-1 study group, H J Adriaanse, E S A van den Akker, M I Baas, C M C Bank, E van Beek, B A de Boer, K de Boer, D M R van der Borden, H A Bremer, J T J Brons, J M Burggraaff, H Ceelie, H Chon, J L M Cikot, F M C Delemarre, J H C Diris, M Doesburg-van Kleffens, I M A van Dooren, J L P van Duijnhoven, F M van Dunné, J J Duvekot, P Engbers, M J W van Etten-van Hulst, H Feitsma, M A Fouraux, M T M Franssen, M A M Frasa, A J van Gammeren, N van Gemund, F van der Graaf, C J M de Groot, C M Hackeng, D P van der Ham, M J C P Hanssen, T H M Hasaart, H A Hendriks, Y M C Henskens, B B J Hermsen, S Hogenboom, A Hooker, F Hudig, A M G Huijssoon, A J M Huisjes, N Jonker, P J Kabel, C van Kampen, M H de Keijzer, D H van de Kerkhof, J F W Keuren, J F W Keuren, G Kleiverda, J H Klinkspoor, S G A Koehorst, M Kok, R D Kok, J B de Kok, A Koops, W Kortlandt, J Langenveld, M P G Leers, A Leyte, A de Mare, G D M Martens, J H Meekers, C A van Meir, G C H Metz, E C H J Michielse, L J Mostert, S W H Nij Bijvank, E Oostenveld, N Osmanovic, M A Oudijk, C Pagano Mirani-Oostdijk, E C M van Pampus, D N M Papatsonis, R H M Peters, G A E Ponjee, M Pontesilli, M M Porath, M S Post, J G J Pouwels, L Prinzen, J M T Roelofsen, J J M Rondeel, P C M van der Salm, H C J Scheepers, D H Schippers, N W E Schuitemaker, J M Sikkema, J Slomp, J W Smit, Y S Snuif-de Lange, J W J van der Stappen, P Steures, G H M Tax, M Treskes, H J L M Ulenkate, G A van Unnik, B S van der Veen, T E M Verhagen, J Versendaal, B Visschers, O Visser, H Visser, K M K de Vooght, M J de Vries, H de Waard, F Weerkamp, M J N Weinans, H de Wet, M van Wijnen, W J van Wijngaarden, A C de Wit, M D Woiski

**Affiliations:** 1 Center for Clinical Transfusion Research, Sanquin Research, Plesmanlaan 1a – 5th floor, 2333 BZ Leiden, The Netherlands; 2 Department of Clinical Epidemiology, Leiden University Medical Center, Albinusdreef 2, 2333 ZA Leiden, The Netherlands; 3 Department of Obstetrics, Leiden University Medical Center, Albinusdreef 2, 2333 ZA Leiden, The Netherlands; 4 National Perinatal Epidemiology Unit, University of Oxford, University of Oxford, Old Road Campus, Oxford, OX3 7LF UK; 5 Department of Obstetrics, Birth Centre Wilhelmina’s Children Hospital, University Medical Center Utrecht, Lundlaan 6, 3584 EA Utrecht, The Netherlands; 6 Athena Institute, VU University Amsterdam, De Boelelaan 1105, 1081 HV Amsterdam, The Netherlands; 7 Department of Internal Medicine, Division of Thrombosis and Haemostasis, Leiden University Medical Center, Leiden, the Netherlands

**Keywords:** Coagulation parameters, Dilutional coagulopathy, Fluid management, Postpartum haemorrhage

## Abstract

**Background:**

The view that 2 l of crystalloid and 1.5 l of colloid can be infused while awaiting compatible blood for patients with major postpartum haemorrhage is based on expert opinion documents. We describe real-world changes in levels of coagulation parameters after the administration of different volumes of clear fluids to women suffering from major postpartum haemorrhage.

**Methods:**

We performed a nationwide retrospective cohort study in the Netherlands among 1038 women experiencing severe postpartum haemorrhage who had received at least four units of red cells or fresh frozen plasma or platelets in addition to red cells. The volume of clear fluids administered before the time of blood sampling was classified into three fluid administration strategies, based on the RCOG guideline: < 2 L, 2–3.5 L and > 3.5 L. Outcomes included haemoglobin, haematocrit, platelet count, fibrinogen, aPTT and PT levels.

**Results:**

Haemoglobin, haematocrit, platelet count, fibrinogen and aPTT were associated with volumes of clear fluids, which was most pronounced early during the course of postpartum haemorrhage. During the earliest phases of postpartum haemorrhage median haemoglobin level was 10.1 g/dl (IQR 8.5–11.6) among the women who received < 2 L clear fluids and 8.1 g/dl (IQR 7.1–8.4) among women who received > 3.5 L of clear fluids; similarly median platelet counts were 181 × 10^9^/litre (IQR 131–239) and 89 × 10^9^/litre (IQR 84–135), aPTT 29 s (IQR 27–33) and 38 s (IQR 35–55) and fibrinogen 3.9 g/L (IQR 2.5–5.2) and 1.6 g/L (IQR 1.3–2.1).

**Conclusions:**

In this large cohort of women with severe postpartum haemorrhage, administration of larger volumes of clear fluids was associated with more severe deterioration of coagulation parameters corresponding to dilution. Our findings provide thus far the best available evidence to support expert opinion-based guidelines recommending restrictive fluid resuscitation in women experiencing postpartum haemorrhage.

**Trial registration:**

Netherlands Trial Register (NTR4079), registration date July 17, 2013.

**Electronic supplementary material:**

The online version of this article (10.1186/s12884-018-2021-9) contains supplementary material, which is available to authorized users.

## Background

Postpartum haemorrhage continues to be a leading cause of maternal health problems worldwide [1]. Depending on the primary cause of haemorrhage, acquired coagulopathy may develop during the course of postpartum haemorrhage and aggravate bleeding [2]. Rapid intravenous infusion of clear (crystalloid and colloid) fluids is generally applied during on-going haemorrhage to establish haemodynamic stability, restore adequate intravascular volume and improve oxygen carrying capacity and oxygen tissue delivery [3]. When given in large volumes, clear fluids initiate dilution of clotting factors resulting in impairment of coagulation and coagulopathy [4–6]. On top of that, rapid consumption of fibrinogen, clotting factors and platelets as a result of persistent blood loss, aggravates coagulopathy [5]. The use of colloid fluids has proven to negatively influence coagulation capacity and endothelial function [7, 8]. These findings have led to less aggressive fluid management in patients with traumatic haemorrhagic shock [9].

International guidelines on management of women with severe postpartum haemorrhage elucidate the lack of quantitative evidence on the effect of different fluid management strategies on parameters of coagulopathy. For instance, the RCOG green-top guideline advises to follow the expert opinion-based recommendation to administer up to 3.5 l of warmed clear fluids, starting with 2 l of warmed isotonic crystalloids until blood products are available in case of persistent postpartum blood loss exceeding 1000 ml [10]. The experts formed their opinions based on experiments in laboratories, animals, healthy volunteers, and observations from trauma patients. However, findings from these studies may not apply to pregnant women, since pregnancy induces haemodynamic and haematologic changes that protect them against haemorrhage during birth. Maternal blood volume increases between 1.2 and 1.6 l above non-pregnant values, creating a hypervolemic state during pregnancy [4]. To enable evidence-based recommendations on fluid management strategies in women with major postpartum haemorrhage, more insight is needed on the changes of coagulation parameters after administration of different volumes of fluids [4]. To the best of our knowledge no previous studies have been conducted into different fluid management strategies and their possible effect on coagulation parameters in women experiencing postpartum haemorrhage.

The aim of this study was to describe the association between administration of different volumes of clear fluids and levels of coagulation parameters in women experiencing postpartum haemorrhage.

## Methods

### Design and study population

We studied volumes of clear fluids and results of coagulation parameter measurements during postpartum haemorrhage in a cohort of women who had been included in a nationwide retrospective cohort study in 61 hospitals in the Netherlands, the TeMpOH-1 (Transfusion strategies in women during Major Obstetric Haemorrhage) study. Included in the TeMpOH-1 study were women who received at least four units of red cells or any transfusion of fresh frozen plasma (FFP) and/or platelets in addition to red cells because of *obstetric haemorrhage* defined as ≥1000 mL blood loss during pregnancy, childbirth or puerperium between January 1st, 2011 and January 1st, 2013. For the present analyses, we selected women from the TeMpOH-1 cohort who met criteria for *primary postpartum haemorrhage*: any amount of blood loss exceeding 1000 mL within the first 24 h after childbirth. Women with no coagulation parameters measured during active postpartum haemorrhage and women with missing data on volumes and timing of clear fluids were excluded. In case transfusion of blood products occurred before onset of clear fluid administration, patients were also excluded. The Ethical Committee of Leiden University Medical Centre (P12.273) and the institutional review boards of all participating hospitals approved of the study. The study was registered in the Netherlands Trial Register (NTR4079). Details regarding study design have been reported elsewhere [11]. The need to obtain informed consent was waived by the ethics committee because of the retrospective design. Women 18 years of age and older who met the inclusion criteria were selected.

### Data collection

To identify all consecutive women who had been transfused with the aforementioned amount of blood products because of postpartum haemorrhage in the participating hospitals, data from the hospitals’ blood transfusion services were merged with data from birth registers of contributing hospitals. Qualified medical students and research nurses collected routine data from the medical records with regard to (obstetric) history and course of the current pregnancy, as well as data pertaining to characteristics of participating women, mode of birth, primary cause of haemorrhage, placentation, characteristics of shock (defined as systolic blood pressure < 90 mmHg or heartrate > 120 bpm), surgical and haemostatic interventions to stop bleeding and coagulation parameters. Results of all measurements of haemoglobin level (Hb, g/dl), haematocrit (Ht, fraction), platelet count (× 10^9^/litre), activated partial thromboplastin time (aPTT, seconds), prothrombin time (PT, seconds) and fibrinogen (g/L) levels from the first measurement of blood loss onwards were documented; this included parameters drawn from cases before they had bled a total volume of 1000 mL. Outliers of levels of coagulation parameters were verified in the medical records. In addition, detailed information on crystalloid and colloid fluids administered during the course of postpartum haemorrhage was collected: total volume and type of clear fluids given, as well as timing information with regard to onset and end of infusion. Information on timing and volume of repetitive blood loss measurements was also retrieved from the medical files. In most cases blood loss was measured by weighing soaked gauzes during and after birth and by use of a collector bag and suction system in the operating theatre.

### Severe acute maternal morbidity and maternal mortality

The composite endpoint severe acute maternal morbidity and mortality comprised emergency peripartum hysterectomy, ligation of the uterine arteries, B-Lynch suture (in the Netherlands only used as emergency procedure), arterial embolization or admission into an intensive care unit.

### Statistical analyses

The aim was to describe values of measured laboratory parameters according to increasing “volume of blood loss” and “volume of clear fluids administered” during the course of severe postpartum haemorrhage. In order to have an estimate of the “volume of blood loss” and of “volume of clear fluids administered” for all blood samples (and their respective laboratory results) we used linear interpolation of the actual measurement of “volume of blood loss” and “volume of clear fluids administered” before and after each blood sample. The volume of blood loss at the time of blood sampling was categorised in 8 groups: 0–1.0 L, 1.0–1.5 L, 1.5–2.0 L, 2.0–2.5 L, 2.5–3.0 L, 3.0–3.5 L, 3.5–4.0 L and > 4.0 L. Coagulation parameters were allocated to the category representing the volume of blood loss at sampling. In case of multiple laboratory measurements per patient within one blood loss category, the mean of the values was used in the analyses, calculating a patient just once per category. Subsequently, within these blood loss categories, the volume of clear fluids administered at the time of blood sampling was calculated and classified into three fluid administration strategies: < 2.0 L, 2.0–3.5 L and > 3.5 L. These three administration strategies were based on the RCOG green-top guideline, which recommends to administer up to 3.5 l of warmed clear fluids, starting with 2 l of warmed isotonic crystalloids if blood is not available [10] . Since blood sampling during postpartum haemorrhage was not performed at predefined time points and samples were obtained on request of the physician on call during postpartum haemorrhage, patients could have different frequencies and panels of coagulation parameters. Reference ranges of aPTT varied somewhat for the 61 participating hospitals as a result of use of different types of reagents. Therefore, an aPTT ratio was calculated by dividing the aPTT level of cases by the mean of the hospital specific reference range.

## Results

### Patient characteristics

**Fig. 1:**
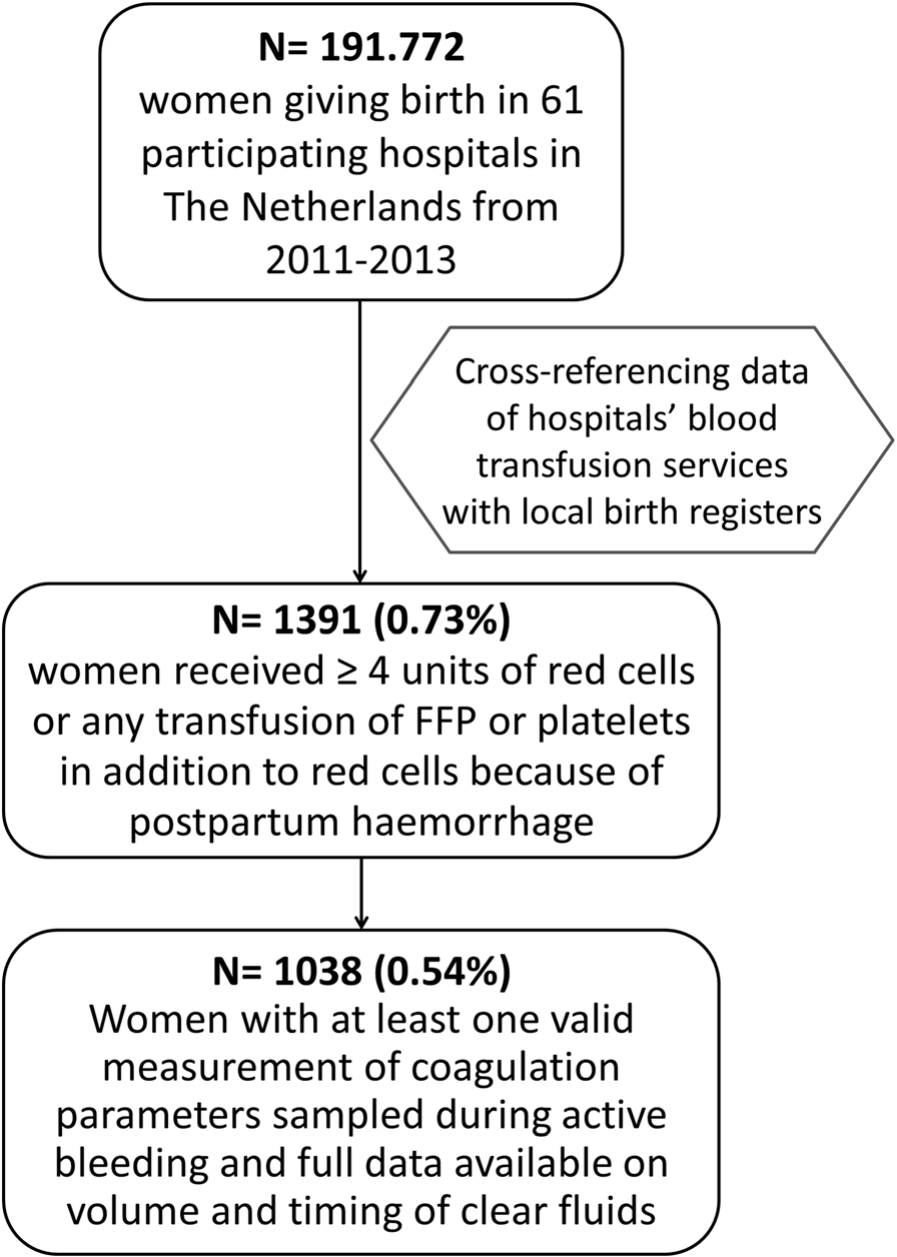
Inclusion flowchart for ‘fluid management and dilutional coagulopathy in severe postpartum haemorrhage: a nationwide retrospective cohort study’

A total of 1038 women with severe postpartum haemorrhage had at least one valid measurement of coagulation parameters sampled during active bleeding in addition to data on volume and timing of clear fluids administered (Fig. 1)*.* Baseline characteristics are reported in Table 1. Women were on average 31 years of age, gave birth at a median gestational age of 39.7 weeks and 25% delivered by caesarean section. Uterine atony was the primary cause of bleeding in 66% of the cases and 34% of women developed a composite endpoint of severe acute maternal morbidity or mortality. The median total volume of blood loss among all 1038 women with postpartum haemorrhage was 3.0 L (interquartile range 2.5–4.0). In our cohort, women in the lowest fluid categories showed fewer signs of shock and were administered fewer blood products when compared to women in the other fluid categories for all coagulation parameters (*data presented in table adjacent to* Fig. 3).

**Table 1:** Clinical characteristics of the cohort of 1038 women with ongoing postpartum haemorrhage included in this analysis

Patients	*n* = 1038
Maternal characteristics	
Age (years)	31.0 (28.0–35.0)^a^
BMI (kg/m^2^)	23.2 (21.0–26.3)
Ethnicity Caucasian	747 (72%)^b^
Nulliparity	534 (51%)
Gestational age	39.7 (38.1–40.7)
Mode of birth	
Caesarean section	254 (24%)
Vaginal	780 (75%)
Comorbidity	
Pre-eclampsia/ HELLP	104 (10%)
Anti-coagulant use	6 (0.6%)
Transfer to hospital	
No transfer (birth in hospital)	753 (73%)
Transfer to hospital during labour	157 (15%)
Postpartum transfer (birth at home)	128 (12%)
Primary cause of bleeding	
Uterine atony	684 (66%)
Retained placenta	168 (16%)
Pathological ingrowth of placenta	89 (9%)
Surgical bleeding and abruption/coagulopathy	97(9%)
Placentation	
Abnormal localisation placenta	65 (6%)
Pathological ingrowth placenta	97 (9%)
Composite endpoint severe maternal morbidity and mortality	355 (34%)
Embolisation	124 (12%)
Hysterectomy	57 (5%)
Emergency B-Lynch	27 (3%)
Ligation arteries	7 (0.7%)
ICU admission	295 (28%)
Maternal mortality	6 (0.6%)
Haemostatic interventions	
Fibrinogen administered	98 (9%)
Tranexamic acid administered	473 (46%)
Recombinant FVIIa administered	29 (3%)
Bleeding characteristics	
Bleeding rate (ml/min) ^c^	2.4 (1.3–4.8)
Shock	927 (89%)
Total volume blood loss (L)	3.0 (2.5–4.0)
Total volume of clear fluids (L)	3.0 (2.0–4.0)
Total units of blood products (n)	6.0 (4.0–8.0)

^a^Values are presented as median with (interquartile range), ^b^percentage, ^c^ maximum

### Volume expansion and volume of blood loss

**Fig. 2:**
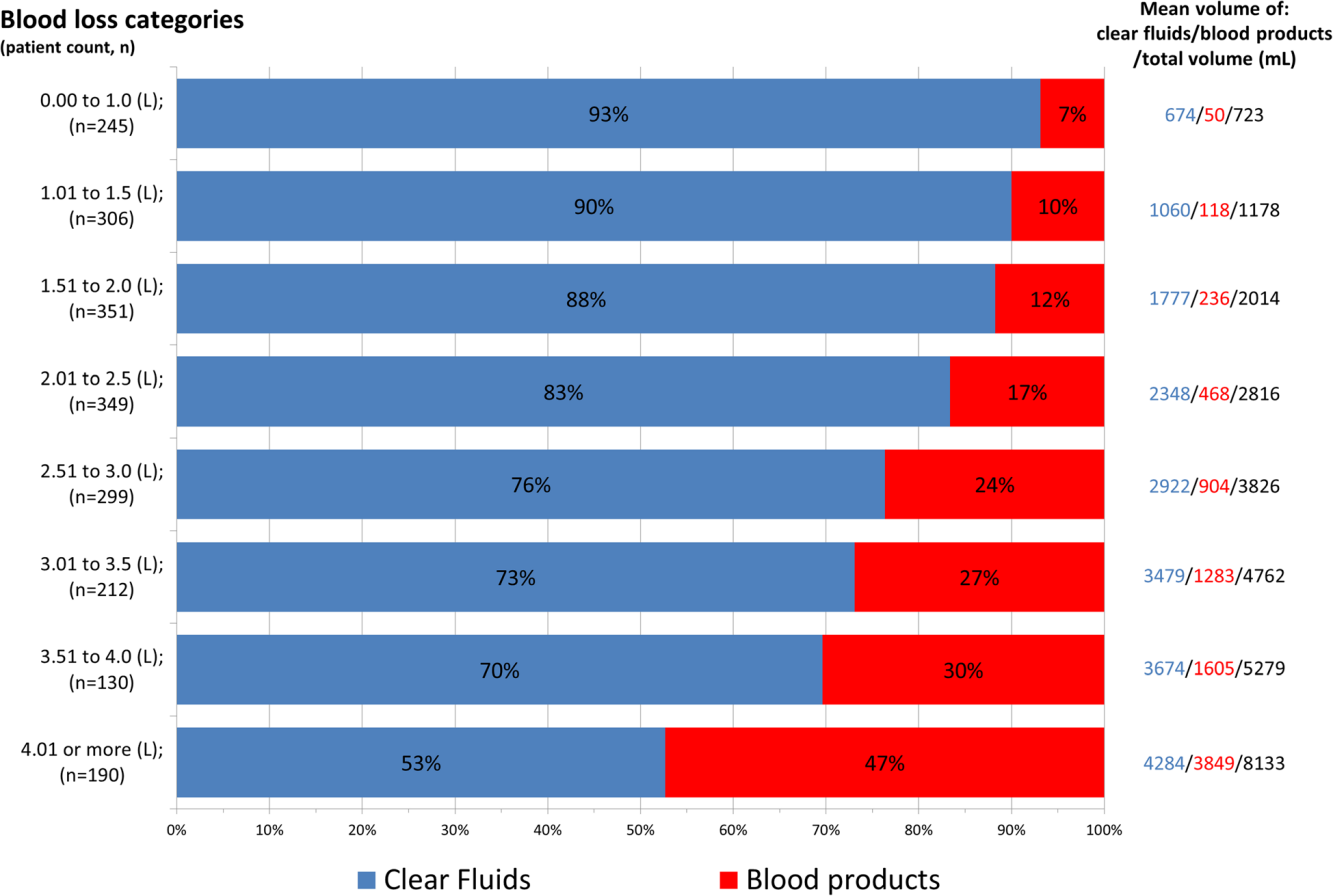
Volume of clear fluids and blood products administered per blood loss category. For example: in the blood loss category 0.0 to 1.0 L 245 women had one or more laboratory parameter tested, and at the time of blood sampling for the laboratory parameters these women had received 674 ml clear fluids, 50 ml blood products, yielding a total volume administered of 723 mL

Figure 2 presents volumes of blood loss and volumes of infused fluids. Among women who had one or more laboratory parameters measured during the first phases of postpartum haemorrhage (*n* = 245 for 0 to 1 L; *n* = 306 for 1 to 1.5 L; and *n* = 351 for 1.5 to 2 L) the mean volume of replacement therapy (clear fluids and blood products) administered was less or equal the total volume of blood loss. During the next phases of postpartum haemorrhage (blood loss between 2 and 2.5 L) the mean volume of replacement therapy (clear fluids and blood products) was higher than the volume of blood loss. This “overload” enlarged with increasing blood loss volumes, reaching 32% more volume replacement compared to blood loss in the phase in which the women had lost 3.5-4 L (5.3 L infused /4 L lost). For all categories of blood loss, mean volume of clear fluids administered did not exceed and in most cases was similar to the maximum blood loss. With increasing blood loss, the proportion of blood products (versus clear fluids) administered showed a gradual increase, from 118/1178 mL (10%) at 1000-1500 mL blood loss to 1605/5279 mL (30%) after blood loss up to 4000 mL.

### Laboratory parameters after different volumes of clear fluids in the course of postpartum haemorrhage

**Fig. 3:**
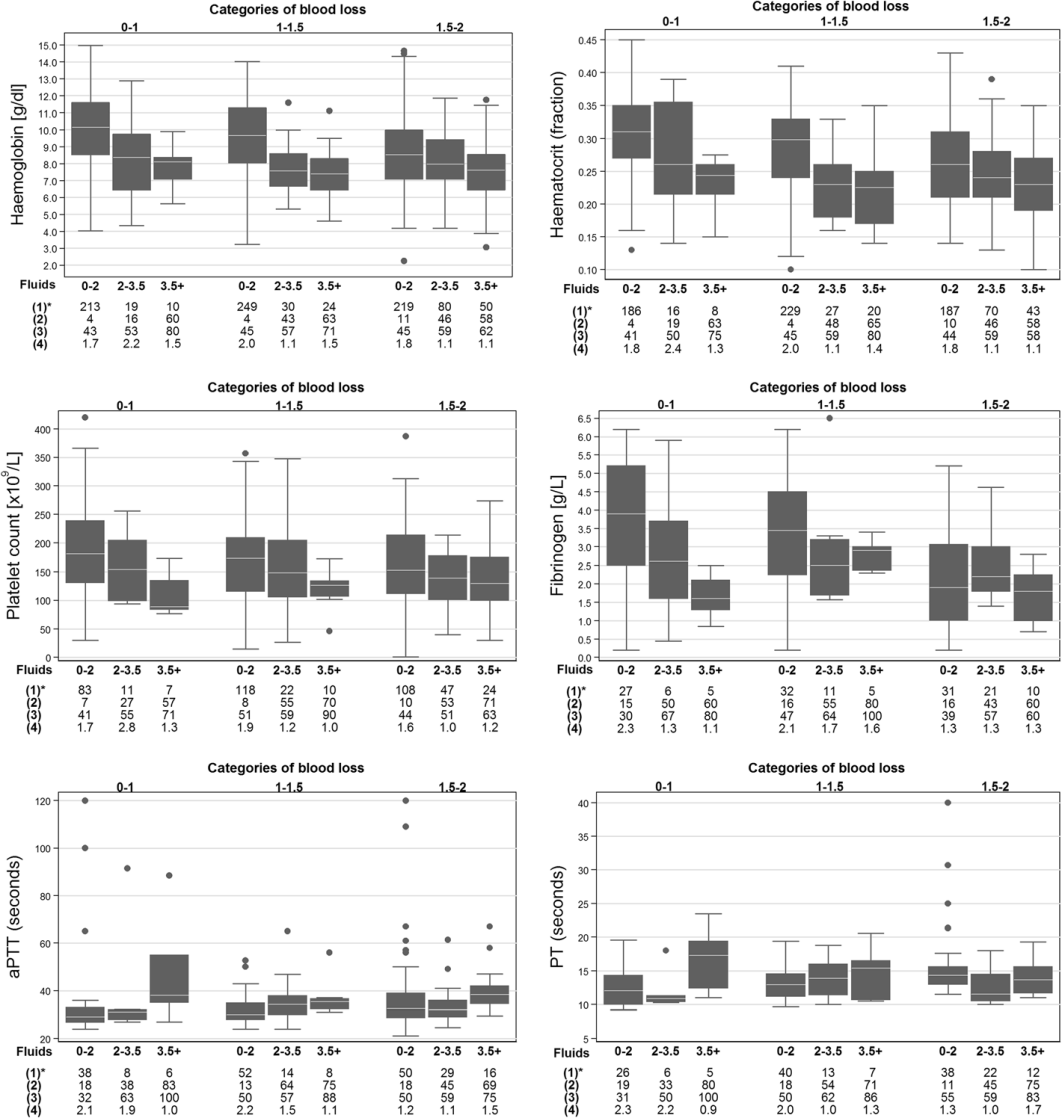
Coagulation parameters according to clear fluid administration (0-2 L, 2 L–3.5 L, > 3.5 L) and increasing volume of blood loss (0–1.0, 1.0–1.5, 1.5–2.0 L). Laboratory parameters are presented in box plots. Circles are outliers. The box represents the 25th and 75th percentiles and the whiskers are the upper and lower adjacent values. *Statistics: (1) Patient count; (2) Percentage of women who received blood products; (3) Percentage of women who experienced shock surrounding blood sampling; (4) mean bleeding rate in ml/min surrounding blood sampling

Figure 3 presents results of laboratory tests according to received volumes of clear fluids (0 to 2 L, 2 to 3.5 L or more than 3.5 L) during the first two litres of postpartum haemorrhage. From 1031 women a total of 2714 haemoglobin measurements were available. Administration of higher volumes of clear fluids was associated with lower haemoglobin and haematocrit levels and this was most pronounced in the earlier phases of postpartum haemorrhage (Fig. 3 and Additional file 1: Table S1 and Additional file 2: Figure S2). For example, when the women had lost less than 1.0 L of blood, the median haemoglobin level was 10.1 g/dl (IQR 8.5–11.6) if they had received < 2.0 L of clear fluids, whereas after receiving 2.0–3.5 L clear fluids median haemoglobin was 8.4 g/dl (IQR 6.4–9.7).

Platelet counts of 804 women decreased over the three increasing fluid administration categories. In samples drawn in the earliest phase of postpartum haemorrhage (0-1 L blood loss), median platelet counts were 181 (IQR 131–239), 154 (IQR 99–205) and 89 × 10^9^/litre (IQR 84–135) in the three categories of increasing volumes of fluids administered. A similar pattern was observed in consecutive blood loss categories.

Fibrinogen measurements of 438 women were available for analyses. Administering higher volumes of clear fluids was associated with a decreasing level of fibrinogen in measurements in the early phases of postpartum haemorrhage (up to 2 L of blood loss). The largest change was displayed for measurements performed in the earliest phase of postpartum haemorrhage (blood loss 0-1000 mL): 3.9 g/L (IQR 2.5–5.2), 2.6 g/L (IQR 1.6–3.7), 1.6 g/L (IQR 1.3–2.1) over the three fluid management categories.

PT and aPTT were longer after administration of larger volumes of clear fluids. For both, the largest difference was observed between measurements in the most restrictive fluids category (< 2 L) and the most liberal category (> 3.5 L). In samples drawn between 0 and 1 L blood loss, PT was 13 (IQR 11–15) and 17 s (IQR 12–19) and aPTT 29 (IQR 27–33) and 38 s (IQR 35–55) in lowest and highest fluid administration categories respectively. Levels of PT and aPTT of women administered 2–3.5 L of fluids were similar to blood samples of women who were administered less than 2 L of fluids. Results of the aPTT ratio showed similar results (Additional file 3: Figure S3).

## Discussion

This nationwide retrospective multicentre cohort study describes coagulation parameters after administering different volumes of resuscitation fluids in 1038 women with ongoing severe postpartum haemorrhage. The administration of larger volumes of clear fluids was associated with deterioration of levels of haemoglobin, haematocrit, platelet count, fibrinogen, aPTT and PT which was most pronounced during the earlier phases of postpartum haemorrhage.

### Strengths and limitations of our study

A strength of the study is that we included a large cohort of women who had suffered severe postpartum haemorrhage and who had been treated with different volume replacement strategies. Women in our study were categorised based on similar volumes of blood loss at time of blood sampling, thereby making them comparable on a clinical level during the course of haemorrhage. Volume replacement had been carefully documented in the medical files in all the participating hospitals ensuring correct classification of women according to the different replacement strategies. Both these strengths allow for reliable description of abnormalities in coagulation in relation to volume replacement therapy.

We stratified our findings according to volume of blood loss. Volume of blood loss was measured in most cases by weighing soaked gauzes during and after birth and by use of a collector bag and suction system in the operating theatre, in addition to visual estimation. Thus, there may be misclassification of volume of blood loss in both directions, over- and underestimation and it is therefore difficult to know whether and how our findings are affected by this misclassification. Our findings are also affected by the fact that inherently more blood samples are drawn from women with more severe bleeding. This may have led to overestimation of the number of women with abnormal laboratory test results. Because of the design of the study we did not have influence on the number and specific panels of coagulation samples requested. Therefore, our results show different selections of women in all blood loss categories that we present. Although it is tempting to infer that high volumes of clear fluids are causally related to the observed dilution our study does not allow such inference. There are many other factors that may have influenced coagulation parameters such as the primary cause of haemorrhage, bleeding and treatment characteristics and the presence of comorbidities. This descriptive study does not allow for disentanglement of the separate effects of these joint risk factors. We excluded 353 women because they had no valid lab measurement available during active bleeding or data were missing on volume or timing of clear fluids administered. To be certain their exclusion did not induce a systemic error to our data resulting from selection bias, we compared these women on the most relevant Table 1 items: mode of birth, nulliparity, primary cause of haemorrhage, the composite endpoint of severe maternal morbidity and mortality, bleeding rate at sampling, presence of shock and total volume of blood loss. No differences were observed compared to the women that were included in the study, ruling out the presence of a systemic error influencing the results.

### Comparison with other studies

To the best of our knowledge no previous studies have described the association between different fluid management strategies and coagulation parameters during the various phases of severe postpartum haemorrhage. Yet, our findings corroborate results of previous studies into the effect of dilution on coagulation parameters. An in vitro study evaluating the effect of haemodilution on coagulation factors found that PT and aPTT were significantly prolonged after 60% and 80% dilution [12]. Another in vitro study investigated the effect of haemodilution on the course of global coagulation tests and clotting factors. Levels of dilution-dependent coagulation factors and aPTT were found to decrease in an almost linear manner. Critically low activities for coagulation factors and a critically low level of fibrinogen were measured at dilutions of between 60 and 75% [13]. An in vivo study reported coagulation parameters in hypotensive patients with penetrating torso injuries who were treated with immediate versus delayed fluid resuscitation. Patients in the immediate fluid administration group showed worse levels of haemoglobin, platelet count, PT and APTT compared to patients in the delayed fluid administration group [14]. No previous studies were found that examined the change in coagulation parameters as a result of different fluid management strategies in women experiencing postpartum haemorrhage.

### Clinical implications

In our cohort of women experiencing postpartum haemorrhage, we displayed changes occurring on coagulation parameter level after administering different volumes of fluids. Administration of larger volumes of clear fluids was associated with more severe worsening of levels of haemoglobin, haematocrit, platelet count, fibrinogen, aPTT and PT which was most pronounced during the earlier phases of postpartum haemorrhage. Our findings provide quantitative evidence to reinforce expert opinion-based guidelines recommending restrictive fluid resuscitation strategies in case of postpartum haemorrhage;

## Conclusions

In this nationwide retrospective cohort study in 1038 women on the change in coagulation parameters with increasing volumes administered during the course of postpartum haemorrhage necessitating blood transfusion, the administration of large volumes of clear fluids was associated with changes in coagulation parameters corresponding to dilutional coagulopathy. Our findings provide thus far the best available evidence to support expert opinion-based guidelines recommending restrictive fluid resuscitation in women experiencing postpartum haemorrhage.

## Additional files

Additional file 1: **Table S1.** Patient count, mean, sd, median and IQR for coagulation parameters in addition to Fig. 3. (DOCX 63 kb)

Additional file 2: **Figure S2.** Coagulation parameters according to clear fluid administration (0-2 L, 2 L–3.5 L, > 3.5 L) and increasing volume of blood loss (0–1.0, 1.0–1.5, 1.5–2.0 L, 2.0–2.5 L, 2.5–3.0 L, 3.0–3.5 L, 3.5–4.0 L and > 4 L). (DOCX 357 kb)

Additional file 3: **Figure S3.** aPTT ratio according to clear fluid administration (0-2000 mL, 2000 mL–3500 mL, > 3500 mL) and increasing blood loss (0–1.0, 1.0–1.5, 1.5–2.0, 2.0–2.5 l). (DOCX 36 kb)

## Abbreviations

aPTT: Activated Partial Thromboplastin Time
Hb: Haemoglobin
Ht: Haematocrit
IQR: Interquartile range
PT: prothrombin time
RCOG: Royal College of Obstetricians and Gynaecologists
