# Supplementary material for: Association between fluid management and dilutional coagulopathy in severe postpartum haemorrhage: a nationwide retrospective cohort study
Source: BMC Pregnancy Childbirth. 2018 Oct 11;18:398. doi: 10.1186/s12884-018-2021-9 (PMC6180574; doi:10.1186/s12884-018-2021-9)
Supplement: Supplementary file 1 — Table S1. Patient count, mean, sd, median and IQR for coagulation parameters in addition to Fig. 3. (DOCX 63 kb) [file 12884_2018_2021_MOESM1_ESM.docx]

## Table S1: Patient count, mean, sd, median and IQR for coagulation parameters in addition to Figure 3

|  |  | **Fluids category** | | | | | | | | | | | | | | | | | | | |
| --- | --- | --- | --- | --- | --- | --- | --- | --- | --- | --- | --- | --- | --- | --- | --- | --- | --- | --- | --- | --- | --- |
|  |  | **0-2(L)** | | | | | |  | **2-3.5(L)** | | | | | |  | **3.5+(L)** | | | | | |
|  | ***Blood loss*** | **n** | **mean** | **sd** | **p50** | **p25** | **p75** | **xx** | **n** | **mean** | **sd** | **p50** | **p25** | **p75** | **xx** | **n** | **mean** | **sd** | **p50** | **p25** | **p75** |
| **Hemoglobin** | |  |  |  |  |  |  |  |  |  |  |  |  |  |  |  |  |  |  |  |  |
|  | 0.00 to 1.0 (L) | 213 | 10.1 | 2.1 | 10.1 | 8.5 | 11.6 |  | 19 | 8.4 | 2.3 | 8.4 | 6.4 | 9.7 |  | 10 | 7.9 | 1.1 | 8.1 | 7.1 | 8.4 |
|  | 1.01 to 1.5 (L) | 249 | 9.6 | 2.1 | 9.7 | 8.1 | 11.3 |  | 30 | 7.6 | 1.5 | 7.6 | 6.7 | 8.6 |  | 24 | 7.4 | 1.6 | 7.4 | 6.4 | 8.3 |
|  | 1.51 to 2.0 (L) | 219 | 8.7 | 2.2 | 8.5 | 7.1 | 10.0 |  | 80 | 8.0 | 1.7 | 8.0 | 7.1 | 9.4 |  | 50 | 7.5 | 1.8 | 7.6 | 6.4 | 8.5 |
|  | 2.01 to 2.5 (L) | 158 | 7.9 | 1.9 | 7.8 | 6.6 | 9.2 |  | 102 | 7.7 | 1.8 | 7.7 | 6.4 | 8.9 |  | 86 | 7.5 | 1.7 | 7.5 | 6.6 | 8.4 |
|  | 2.51 to 3.0 (L) | 76 | 7.7 | 1.6 | 7.8 | 6.4 | 8.7 |  | 105 | 7.6 | 1.7 | 7.6 | 6.4 | 8.7 |  | 107 | 7.7 | 1.7 | 7.9 | 6.4 | 8.9 |
|  | 3.01 to 3.5 (L) | 37 | 8.0 | 1.7 | 8.1 | 6.8 | 8.7 |  | 69 | 8.1 | 1.9 | 8.1 | 6.9 | 9.3 |  | 102 | 8.0 | 1.7 | 8.1 | 6.8 | 9.0 |
|  | 3.51 to 4.0 (L) | 23 | 8.7 | 2.0 | 9.3 | 7.4 | 9.8 |  | 42 | 8.2 | 1.6 | 8.5 | 7.1 | 9.4 |  | 65 | 8.0 | 1.7 | 8.2 | 7.1 | 9.0 |
|  | 4.01 or more (L) | 27 | 9.1 | 2.0 | 8.7 | 7.7 | 10.5 |  | 50 | 8.2 | 1.5 | 8.1 | 7.2 | 8.9 |  | 110 | 8.4 | 1.7 | 8.3 | 7.2 | 9.5 |
|  | Total | 1002 | 9.0 | 2.2 | 8.9 | 7.4 | 10.6 |  | 497 | 7.9 | 1.7 | 7.9 | 6.8 | 9.0 |  | 554 | 7.9 | 1.7 | 7.9 | 6.8 | 8.9 |
| **Hematocrit** | |  |  |  |  |  |  |  |  |  |  |  |  |  |  |  |  |  |  |  |  |
|  | 0.00 to 1.0 (L) | 186 | 0.31 | 0.06 | 0.31 | 0.27 | 0.35 |  | 16 | 0.27 | 0.08 | 0.26 | 0.21 | 0.36 |  | 8 | 0.23 | 0.04 | 0.24 | 0.21 | 0.26 |
|  | 1.01 to 1.5 (L) | 229 | 0.29 | 0.06 | 0.30 | 0.24 | 0.33 |  | 27 | 0.23 | 0.05 | 0.23 | 0.18 | 0.26 |  | 20 | 0.22 | 0.06 | 0.23 | 0.17 | 0.25 |
|  | 1.51 to 2.0 (L) | 187 | 0.26 | 0.06 | 0.26 | 0.21 | 0.31 |  | 70 | 0.24 | 0.05 | 0.24 | 0.21 | 0.28 |  | 43 | 0.23 | 0.05 | 0.23 | 0.19 | 0.27 |
|  | 2.01 to 2.5 (L) | 132 | 0.24 | 0.06 | 0.24 | 0.20 | 0.28 |  | 82 | 0.24 | 0.05 | 0.24 | 0.20 | 0.27 |  | 73 | 0.23 | 0.05 | 0.23 | 0.20 | 0.26 |
|  | 2.51 to 3.0 (L) | 62 | 0.24 | 0.05 | 0.23 | 0.20 | 0.26 |  | 90 | 0.23 | 0.05 | 0.22 | 0.19 | 0.26 |  | 98 | 0.23 | 0.05 | 0.23 | 0.19 | 0.27 |
|  | 3.01 to 3.5 (L) | 33 | 0.24 | 0.05 | 0.24 | 0.21 | 0.26 |  | 56 | 0.25 | 0.05 | 0.25 | 0.21 | 0.28 |  | 84 | 0.24 | 0.05 | 0.24 | 0.21 | 0.27 |
|  | 3.51 to 4.0 (L) | 21 | 0.27 | 0.06 | 0.28 | 0.25 | 0.30 |  | 38 | 0.24 | 0.05 | 0.23 | 0.20 | 0.27 |  | 53 | 0.23 | 0.05 | 0.24 | 0.20 | 0.26 |
|  | 4.01 or more (L) | 25 | 0.28 | 0.07 | 0.26 | 0.22 | 0.34 |  | 42 | 0.24 | 0.04 | 0.24 | 0.20 | 0.26 |  | 101 | 0.25 | 0.05 | 0.25 | 0.21 | 0.28 |
|  | Total | 875 | 0.27 | 0.06 | 0.27 | 0.22 | 0.32 |  | 421 | 0.24 | 0.05 | 0.24 | 0.20 | 0.27 |  | 480 | 0.24 | 0.05 | 0.24 | 0.20 | 0.27 |
| **Platelet count** | |  |  |  |  |  |  |  |  |  |  |  |  |  |  |  |  |  |  |  |  |
|  | 0.00 to 1.0 (L) | 83 | 184 | 80 | 181 | 131 | 239 |  | 11 | 163 | 59 | 154 | 99 | 205 |  | 7 | 111 | 37 | 89 | 84 | 135 |
|  | 1.01 to 1.5 (L) | 118 | 167 | 72 | 173 | 116 | 209 |  | 22 | 159 | 74 | 148 | 106 | 205 |  | 10 | 123 | 36 | 126 | 107 | 134 |
|  | 1.51 to 2.0 (L) | 108 | 164 | 72 | 153 | 112 | 214 |  | 47 | 137 | 48 | 139 | 101 | 178 |  | 24 | 136 | 56 | 130 | 100 | 175 |
|  | 2.01 to 2.5 (L) | 84 | 164 | 58 | 167 | 125 | 190 |  | 60 | 147 | 68 | 133 | 98 | 181 |  | 50 | 130 | 42 | 133 | 97 | 154 |
|  | 2.51 to 3.0 (L) | 59 | 151 | 55 | 141 | 123 | 181 |  | 75 | 133 | 61 | 122 | 96 | 158 |  | 73 | 125 | 47 | 119 | 93 | 158 |
|  | 3.01 to 3.5 (L) | 20 | 137 | 41 | 122 | 111 | 168 |  | 47 | 124 | 43 | 125 | 94 | 149 |  | 67 | 120 | 52 | 105 | 86 | 146 |
|  | 3.51 to 4.0 (L) | 17 | 131 | 47 | 124 | 100 | 159 |  | 34 | 119 | 50 | 117 | 91 | 139 |  | 46 | 114 | 39 | 112 | 88 | 139 |
|  | 4.01 or more (L) | 22 | 94 | 41 | 83 | 70 | 114 |  | 47 | 108 | 40 | 111 | 79 | 134 |  | 100 | 103 | 39 | 96 | 77 | 122 |
|  | Total | 511 | 161 | 69 | 159 | 112 | 203 |  | 343 | 132 | 57 | 124 | 94 | 160 |  | 377 | 118 | 46 | 110 | 88 | 145 |
| **Fibrinogen** | |  |  |  |  |  |  |  |  |  |  |  |  |  |  |  |  |  |  |  |  |
|  | 0.00 to 1.0 (L) | 27 | 3.5 | 1.8 | 3.9 | 2.5 | 5.2 |  | 6 | 2.8 | 1.9 | 2.6 | 1.6 | 3.7 |  | 5 | 1.7 | 0.6 | 1.6 | 1.3 | 2.1 |
|  | 1.01 to 1.5 (L) | 32 | 3.4 | 1.4 | 3.5 | 2.3 | 4.5 |  | 11 | 2.7 | 1.4 | 2.5 | 1.7 | 3.2 |  | 5 | 2.8 | 0.5 | 2.9 | 2.4 | 3.0 |
|  | 1.51 to 2.0 (L) | 31 | 2.2 | 1.4 | 1.9 | 1.0 | 3.1 |  | 21 | 2.5 | 0.9 | 2.2 | 1.8 | 3.0 |  | 10 | 1.7 | 0.7 | 1.8 | 1.0 | 2.3 |
|  | 2.01 to 2.5 (L) | 35 | 2.2 | 0.9 | 2.1 | 1.8 | 2.8 |  | 25 | 2.3 | 0.6 | 2.5 | 1.9 | 2.7 |  | 22 | 1.8 | 0.6 | 1.7 | 1.3 | 2.3 |
|  | 2.51 to 3.0 (L) | 24 | 2.2 | 0.8 | 1.9 | 1.7 | 2.8 |  | 39 | 2.1 | 0.8 | 2.0 | 1.6 | 2.6 |  | 40 | 2.0 | 0.9 | 1.9 | 1.3 | 2.6 |
|  | 3.01 to 3.5 (L) | 8 | 2.1 | 0.6 | 2.0 | 1.7 | 2.3 |  | 23 | 2.0 | 0.7 | 2.0 | 1.6 | 2.5 |  | 40 | 2.1 | 0.7 | 2.0 | 1.5 | 2.6 |
|  | 3.51 to 4.0 (L) | 11 | 2.7 | 1.2 | 2.3 | 2.0 | 3.3 |  | 20 | 1.8 | 0.8 | 1.7 | 1.5 | 2.0 |  | 25 | 1.6 | 0.5 | 1.6 | 1.3 | 1.8 |
|  | 4.01 or more (L) | 18 | 2.1 | 0.7 | 2.0 | 1.7 | 2.5 |  | 31 | 1.8 | 0.5 | 1.7 | 1.5 | 2.2 |  | 62 | 1.8 | 0.7 | 1.7 | 1.3 | 2.2 |
|  | Total | 186 | 2.6 | 1.4 | 2.2 | 1.7 | 3.4 |  | 176 | 2.1 | 0.9 | 2.0 | 1.6 | 2.6 |  | 209 | 1.9 | 0.7 | 1.8 | 1.3 | 2.3 |
| **PT** | |  |  |  |  |  |  |  |  |  |  |  |  |  |  |  |  |  |  |  |  |
|  | 0.00 to 1.0 (L) | 26 | 13 | 3 | 12 | 10 | 14 |  | 6 | 12 | 3 | 11 | 10 | 11 |  | 5 | 17 | 5 | 17 | 12 | 19 |
|  | 1.01 to 1.5 (L) | 40 | 13 | 2 | 13 | 11 | 15 |  | 13 | 14 | 3 | 14 | 11 | 16 |  | 7 | 15 | 4 | 15 | 11 | 17 |
|  | 1.51 to 2.0 (L) | 38 | 16 | 6 | 14 | 13 | 16 |  | 22 | 13 | 2 | 12 | 11 | 14 |  | 12 | 14 | 3 | 14 | 12 | 16 |
|  | 2.01 to 2.5 (L) | 44 | 15 | 5 | 15 | 12 | 16 |  | 29 | 15 | 4 | 14 | 12 | 16 |  | 26 | 16 | 6 | 15 | 11 | 18 |
|  | 2.51 to 3.0 (L) | 38 | 14 | 3 | 14 | 12 | 16 |  | 57 | 15 | 3 | 14 | 12 | 17 |  | 40 | 15 | 4 | 14 | 11 | 16 |
|  | 3.01 to 3.5 (L) | 12 | 13 | 2 | 13 | 12 | 15 |  | 29 | 14 | 3 | 14 | 12 | 16 |  | 44 | 14 | 2 | 15 | 13 | 16 |
|  | 3.51 to 4.0 (L) | 15 | 16 | 7 | 15 | 12 | 16 |  | 24 | 16 | 6 | 15 | 12 | 17 |  | 29 | 15 | 5 | 14 | 12 | 18 |
|  | 4.01 or more (L) | 17 | 17 | 6 | 15 | 13 | 17 |  | 35 | 15 | 3 | 15 | 12 | 17 |  | 74 | 16 | 7 | 14 | 12 | 18 |
|  | Total | 230 | 15 | 5 | 14 | 12 | 16 |  | 215 | 14 | 4 | 14 | 12 | 17 |  | 237 | 15 | 5 | 14 | 12 | 17 |
| **APTT** | |  |  |  |  |  |  |  |  |  |  |  |  |  |  |  |  |  |  |  |  |
|  | 0.00 to 1.0 (L) | 38 | 34 | 19 | 29 | 27 | 33 |  | 8 | 38 | 22 | 31 | 28 | 32 |  | 6 | 47 | 22 | 38 | 35 | 55 |
|  | 1.01 to 1.5 (L) | 52 | 32 | 6 | 30 | 28 | 35 |  | 14 | 36 | 10 | 34 | 30 | 38 |  | 8 | 37 | 8 | 35 | 32 | 37 |
|  | 1.51 to 2.0 (L) | 50 | 38 | 19 | 32 | 29 | 39 |  | 29 | 34 | 8 | 32 | 29 | 36 |  | 16 | 40 | 10 | 38 | 35 | 42 |
|  | 2.01 to 2.5 (L) | 51 | 39 | 16 | 35 | 31 | 43 |  | 36 | 33 | 6 | 33 | 30 | 36 |  | 35 | 38 | 13 | 33 | 30 | 42 |
|  | 2.51 to 3.0 (L) | 43 | 35 | 9 | 33 | 29 | 37 |  | 59 | 37 | 10 | 35 | 30 | 41 |  | 59 | 38 | 14 | 35 | 31 | 42 |
|  | 3.01 to 3.5 (L) | 13 | 34 | 5 | 33 | 31 | 38 |  | 36 | 35 | 7 | 35 | 31 | 38 |  | 57 | 37 | 10 | 36 | 29 | 41 |
|  | 3.51 to 4.0 (L) | 14 | 40 | 24 | 33 | 28 | 39 |  | 27 | 41 | 21 | 34 | 31 | 40 |  | 34 | 38 | 9 | 37 | 34 | 41 |
|  | 4.01 or more (L) | 19 | 39 | 10 | 36 | 31 | 42 |  | 40 | 39 | 11 | 36 | 32 | 41 |  | 81 | 44 | 22 | 39 | 33 | 46 |
|  | Total | 280 | 36 | 15 | 32 | 29 | 37 |  | 249 | 36 | 11 | 34 | 30 | 39 |  | 296 | 40 | 16 | 36 | 31 | 43 |
| **APTT rate** | |  |  |  |  |  |  |  |  |  |  |  |  |  |  |  |  |  |  |  |  |
|  | 0.00 to 1.0 (L) | 36 | 1.21 | 0.73 | 1.00 | 0.91 | 1.12 |  | 8 | 1.31 | 0.77 | 1.07 | 1.00 | 1.12 |  | 5 | 1.68 | 0.77 | 1.27 | 1.18 | 1.90 |
|  | 1.01 to 1.5 (L) | 46 | 1.09 | 0.26 | 1.02 | 0.92 | 1.17 |  | 12 | 1.24 | 0.32 | 1.17 | 1.06 | 1.31 |  | 8 | 1.27 | 0.26 | 1.16 | 1.13 | 1.29 |
|  | 1.51 to 2.0 (L) | 43 | 1.35 | 0.67 | 1.16 | 0.97 | 1.45 |  | 24 | 1.17 | 0.28 | 1.15 | 0.95 | 1.24 |  | 15 | 1.38 | 0.38 | 1.31 | 1.18 | 1.38 |
|  | 2.01 to 2.5 (L) | 41 | 1.39 | 0.54 | 1.21 | 1.07 | 1.49 |  | 31 | 1.13 | 0.22 | 1.14 | 0.96 | 1.28 |  | 33 | 1.34 | 0.45 | 1.21 | 1.04 | 1.43 |
|  | 2.51 to 3.0 (L) | 36 | 1.21 | 0.35 | 1.16 | 1.01 | 1.31 |  | 55 | 1.28 | 0.36 | 1.20 | 1.04 | 1.36 |  | 55 | 1.30 | 0.48 | 1.19 | 1.03 | 1.45 |
|  | 3.01 to 3.5 (L) | 12 | 1.21 | 0.27 | 1.13 | 1.02 | 1.40 |  | 33 | 1.20 | 0.20 | 1.23 | 1.07 | 1.37 |  | 54 | 1.24 | 0.35 | 1.21 | 0.98 | 1.40 |
|  | 3.51 to 4.0 (L) | 13 | 1.17 | 0.35 | 1.05 | 0.93 | 1.42 |  | 22 | 1.31 | 0.53 | 1.20 | 1.04 | 1.37 |  | 31 | 1.31 | 0.32 | 1.27 | 1.17 | 1.48 |
|  | 4.01 or more (L) | 17 | 1.34 | 0.36 | 1.24 | 1.13 | 1.41 |  | 38 | 1.35 | 0.38 | 1.29 | 1.13 | 1.44 |  | 73 | 1.50 | 0.73 | 1.31 | 1.16 | 1.53 |
|  | Total | 244 | 1.25 | 0.51 | 1.10 | 0.95 | 1.33 |  | 223 | 1.25 | 0.36 | 1.18 | 1.04 | 1.35 |  | 274 | 1.36 | 0.52 | 1.25 | 1.06 | 1.46 |
