# Supplementary material for: Association between fluid management and dilutional coagulopathy in severe postpartum haemorrhage: a nationwide retrospective cohort study
Source: BMC Pregnancy Childbirth. 2018 Oct 11;18:398. doi: 10.1186/s12884-018-2021-9 (PMC6180574; doi:10.1186/s12884-018-2021-9)
Supplement: Supplementary file 2 — Figure S2. Coagulation parameters according to clear fluid administration (0-2 L, 2 L–3.5 L, > 3.5 L) and increasing volume of blood loss (0–1.0, 1.0–1.5, 1.5–2.0 L, 2.0–2.5 L, 2.5–3.0 L, 3.0–3.5 L, 3.5–4.0 L and > 4 L). (DOCX 357 kb) [file 12884_2018_2021_MOESM2_ESM.docx]

## Figure S2: Coagulation parameters according to clear fluid administration (0-2L, 2L-3.5L, >3.5L) and increasing volume of blood loss (0-1.0, 1.0-1.5, 1.5-2.0 L, 2.0-2.5L, 2.5-3.0L, 3.0-3.5L, 3.5-4.0L and >4L).


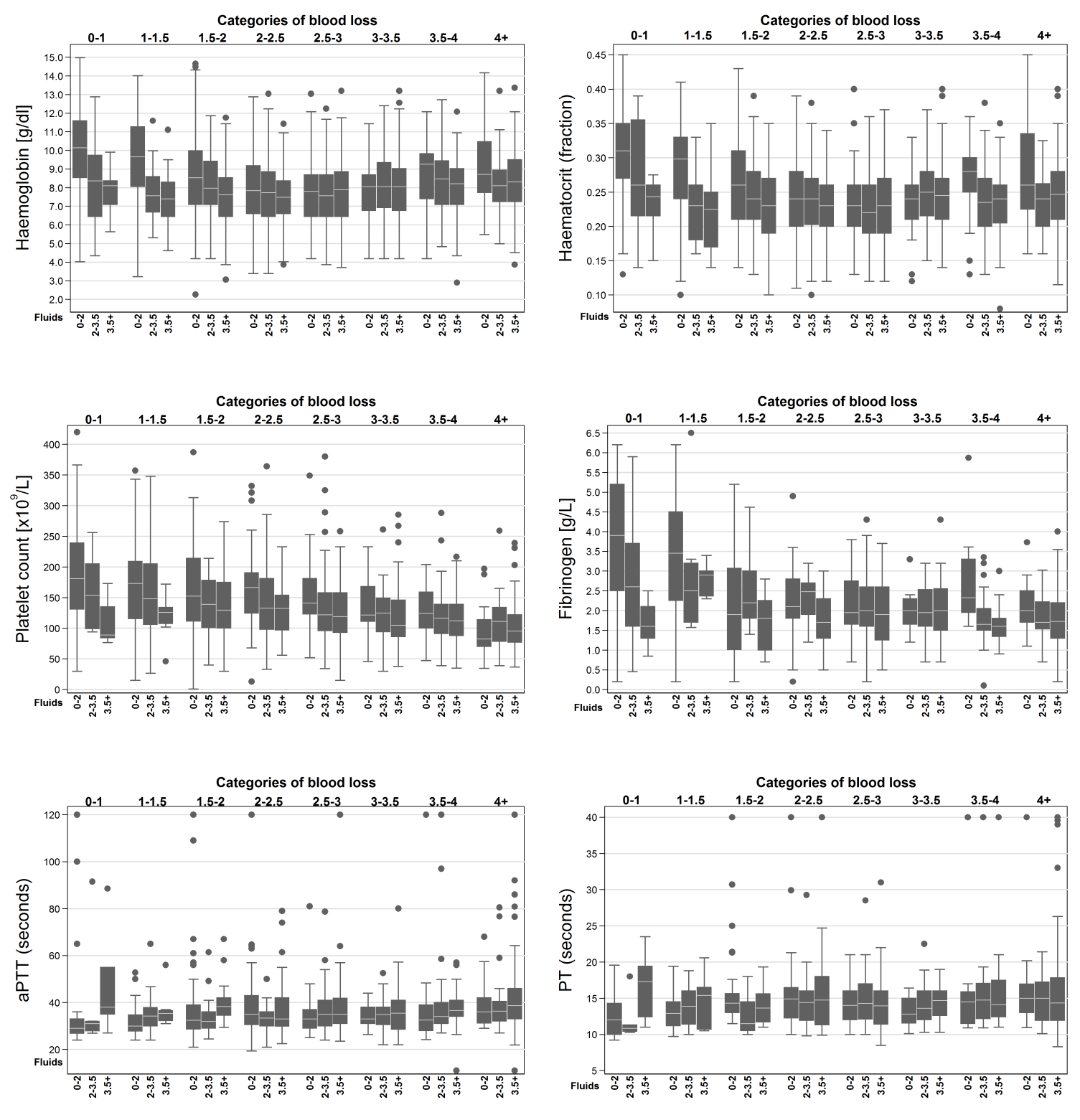


Laboratory parameters are presented in box plots. Circles are outliers. The box represents the 25th and 75th percentiles and the whiskers are the upper and lower adjacent values.
