# Supplementary material for: Association between fluid management and dilutional coagulopathy in severe postpartum haemorrhage: a nationwide retrospective cohort study
Source: BMC Pregnancy Childbirth. 2018 Oct 11;18:398. doi: 10.1186/s12884-018-2021-9 (PMC6180574; doi:10.1186/s12884-018-2021-9)
Supplement: Supplementary file 3 — Figure S3. aPTT ratio according to clear fluid administration (0-2000 mL, 2000 mL–3500 mL, > 3500 mL) and increasing blood loss (0–1.0, 1.0–1.5, 1.5–2.0, 2.0–2.5 l). (DOCX 36 kb) [file 12884_2018_2021_MOESM3_ESM.docx]

*Figure S3 aPTT ratio according to clear fluid administration (0-2000mL, 2000mL-3500mL, >3500mL) and increasing blood loss (0-1.0, 1.0-1.5, 1.5-2.0, 2.0-2.5 l)*

*Statistics: (1) Patient count; (2) Percentage of women who received blood products; (3) Percentage of women who experienced shock surrounding blood sampling; (4) mean bleeding rate in ml/min surrounding blood sampling.
